# Systemic evaluation and optimisation strategies for the synergy of high-value medical consumables policies in China

**DOI:** 10.1080/20523211.2026.2673696

**Published:** 2026-07-31

**Authors:** Huitong Yang, Shuzhen Chu

**Affiliations:** School of International Pharmaceutical Business, China Pharmaceutical University, Nanjing, People’s Republic of China

**Keywords:** High-value medical consumables, centralised procurement, policy synergy, strategy optimisation, three-dimensional analysis framework

## Abstract

**Background:**

The excessively high prices of High-Value Medical Consumables (HVMCs) and the substantial financial burden on patients are core issues in China’s healthcare reform. Although the Volume-Based Procurement (VBP) policies have significantly reduced prices, insufficient policy coordination has led to frequent implementation deviations, constraining the effectiveness of reforms.

**Methods:**

Guided by a three-dimensional framework encompassing policy intensity, measures, and objectives, this study employed an integrated analytical process. Text mining facilitated initial thematic identification, which was then iteratively refined and validated through the Delphi method to establish quantifiable indicators within the framework. These indicators were analysed using a synergy degree model to evaluate policy alignment. 21 national-level policies from 2004 to 2024 are analysed.

**Results:**

Based on the constructed synergy indices, policy development underwent three stages: ‘initial exploration, gradual development, and stable optimization'. As indicated by the model, the synergy degree showed an evolutionary trend, moving from early dispersion, to mid-term leap, and later differentiation. According to the indices, planning guidance and administrative measures showed relatively high synergy over the long term but appeared to squeeze the synergy space for other measures. The results also indicated that imbalances existed among policy objectives, with contradictions between cost control and innovation goals, and insufficient adaptation between medical insurance payment and industry development. Based on these model-based findings, optimisation strategies such as classified coordination mechanisms, dynamic weight adjustment, and closed-loop feedback for quality and supply are proposed.

**Conclusion:**

Theoretically, this study is the first to systematically quantify the degree of synergy of high-value medical consumables policies by developing a framework-based coordination analysis model using constructed indices. Practically, it provides a scientific basis for optimising policy combinations, balancing short-term cost control with long-term industrial innovation, and improving medical insurance support mechanisms.

## 1. Introduction

High-value medical consumables (HVMCs) feature stringent safety requirements, high clinical usage, and substantial costs. Critical for enhancing diagnostic and treatment outcomes, they also impose heavy financial burdens on patients. The OECD (2024) highlights medical device supply chain vulnerabilities, emphasising that national policy effectiveness relies on cross-organisational coordination and private sector collaboration. China’s volume-based procurement (VBP) policies have effectively reduced prices, but insufficient systemic policy coordination has led to unintended challenges. Misaligned medical insurance payment standards, low adoption rates of procured products, and fragmented cross-agency and cross-stage collaboration have undermined reform impact.

Previous research in China has primarily focused on implementation mechanisms and effectiveness evaluation, failing to capture the dynamic synergies across policy phases and between departments, thereby creating a research gap. A limited number of scholars have employed policy tool frameworks (Wu & Chang, 2025). Research on policy synergy across multiple domains has been conducted (Zhang et al., 2025; Dong & Wang, 2025), utilising qualitative (Cao & Li, 2020; Wang & Jin, 2021) and quantitative approaches (Si, 2008; Ma, 2000; Su et al., 2012), yet it lacks depth in unravelling the internal logic and dynamic evolution of policy texts. Internationally, studies emphasise health technology assessment and diverse procurement models (Chen & Huang, 2016), while highlighting transparency and incentive gaps (Cangelosi et al., 2023). The WHO (2023) stresses that global medical procurement effectiveness depends on transparency, supply chain stability, and cross-organisational cooperation. However, international policy synergy frameworks are grounded in Western institutional contexts, offering limited insight into China’s VBP mechanism. No existing study has established a quantifiable framework integrating policy intensity, measures, and objectives to evaluate HVMCs policy synergy.

To address these limitations, this study analyses 21 national-level HVMC VBP policies from 2004 to 2024. The analysis integrates text mining (word frequency and semantic network analysis) with a three-dimensional framework of ‘policy intensity–policy measures–policy objectives'. This framework is rooted in policy coordination theory, where policy intensity maps to execution strength, policy measures to action alignment, and policy objectives to goal coherence. The study then applies the Delphi method and synergy modelling to quantitatively evaluate inter-policy coordination and its evolution. This framework bridges theory and practice, systemically measuring cross-agency and cross-stage synergy to address key gaps. The findings provide a scientific basis for designing coherent, sustainable procurement policies and for enhancing tripartite medical system coordination among medical services, insurance, and pharmaceutical reforms.

## 2. Data and methods

This study adopts a mixed methods framework integrating text mining, the Delphi method and synergy modelling to ensure methodological rigour, transparency and replicability.

### 2.1. Data sources

Using three key search terms ‘centralized procurement of high-value medical consumables', ‘concentrated procurement of high-value medical consumables', and ‘volume-based centralized procurement of high-value medical consumables' relevant documents were retrieved from the CNKI and PKULAW and national government official websites up to April 2025, yielding 217 initial results.

Documents were processed through three systemic steps: keyword-targeted search, result aggregation, and duplicate removal by title and issuing body.

### 2.2. Selection criteria

This study selected national-level policies issued by central government entities, including the State Council and National Healthcare Security Administration NHSA. Eligible policies must be directly related to HVMC VBP with concrete measures or objectives and belong to authoritative formal types such as notices and opinions.

Policies were excluded if they lacked relevance to HVMC VBP, were non-formal documents like reply letters, or had incomplete content.

After systemic screening, 21 valid policies issued between 2004 and 2024 were finalised for the study (see Supplemental Table S1 for complete details).

### 2.3. Research methods

The entire analysis was driven by the three-dimensional analytical framework, providing the foundational structure for qualitative categorisation and quantitative scoring.

#### 2.3.1. Text mining

ROST Content Mining System (Version 6.0, ROST EA 1.9.0.4) was used for analysing 21 policy texts, with key steps:

1. Text cleaning: Removed punctuation, headers/footers, and special symbols; converted to plain text.
2. Stop-word processing: Applied a custom list to exclude non-informative terms (e.g. ‘notice', ‘department').
3. Segmentation & frequency analysis: Used built-in tools for word segmentation and core keyword identification.
4. Manual validation: Independent sorting → team cross-validation → group discussion for ambiguity resolution.
5. Semantic network analysis: Constructed with keywords of frequency ≥10.
6. Missing data handling: Unmentioned indicators labelled ‘not mentioned' and coded 0; no missing expert scores (pre-communication guaranteed).

#### 2.3.2. Delphi method

Complementary to text mining for indicator optimisation (parallel design, no causal relationship):

- Expert panel: 9 cross-disciplinary experts (3 academia, 2 medical institutions, 2 medical insurance, 2 enterprises) with ≥5 years of relevant experience, recruited via academic networks and supervisor coordination.
- Consent and confidentiality: Verbal informed consent was obtained; expert personal information is kept confidential; policies are publicly available official documents.
- Scoring & validity: 1–5 scale (1 = weak/ambiguous, 5 = strong/clear) based on policy clarity and intensity (consistent with policy evaluation norms); weights determined by expert consensus. Content validity ensured by cross-disciplinary coverage; process validity by standardised procedures.
- Procedure: One round of anonymous online scoring was conducted first. After collecting the scores, any disagreements (defined as a score difference of ≥2 points between experts on the same indicator) were identified. This was followed by two rounds of offline facilitated discussions. In each discussion round, experts with divergent views were asked to explain their rationale, and the group attempted to reach consensus through moderated dialogue. If consensus could not be reached after the second discussion round, the final score was determined by the median of the expert scores. The process was finalised via expert consensus, ensuring stable group judgment.

Guided by the three-dimensional framework, text mining identified preliminary categories from policy texts, while the Delphi method validated their relevance. Quantitative scoring directly reflected policy intensity (higher scores = stronger implementation willingness). The synergy model integrated dimension scores to measure alignment between policy measures and objectives, converting qualitative text to quantifiable data.

#### 2.3.3. Conceptual justification

The three-dimensional framework (‘policy intensity–policy measures–policy objectives') is rooted in policy coordination theory and synergy degree theory, evolving from the policy coordination degree model (Peng et al., 2008) while addressing key limitations to fit China’s healthcare governance context.

Compared with mainstream policy synergy models, this framework’s novelty lies in two core improvements: first, it supplements the lack of quantitative linkage between governance capacity and policy text attributes in existing models – most focus on either coordination capacity or tool combinations, while this framework quantifies ‘policy intensity' to reflect administrative authority and resource support, bridging the gap between policy design and implementation strength. Second, it strengthens the synergistic logic between ‘measures' and ‘objectives', which is overlooked in models emphasising vertical hierarchy interactions, to adapt to China’s hybrid governance characteristics.

For context-specific adaptation, the framework aligns with China’s governance reality of ‘strong top-down leadership plus cross-sectoral coordination’: it quantifies administrative hierarchy (e.g. State Council documents = 5 points) and multi-ministerial joint issuance to reflect top-down authority, while linking ‘policy measures' (e.g. regulatory safeguards) and ‘objectives' (e.g. industrial innovation) to address cross-sectoral coordination gaps in ‘tripartite medical reform' (medical services, insurance, pharmaceuticals). This adaptation relies on inherent characteristics of China’s policy system, without relying on external theoretical references.

### 2.4. Quantification standards

An indicator classification and quantitative scoring table for the coordination degree of HVMC VBP policies was constructed in Table 1. The three dimensions of ‘policy intensity–policy measure–policy objective' were selected based on the internal logic of policy system operation and the understanding of coordination essence. The classification primarily focuses on the complete chain of policy effectiveness, revealing core nodes of coordination.

**Table 1.** Policy intensity scoring.

Score	Policy intensity description
5	Policies, regulations, and notices issued by the State Council
4	Policy documents jointly issued by multiple departments
3	Policy documents issued by the National Health Commission
2	Decisions and opinions issued by the National Healthcare Security Administration (NHSA)
1	Notices issued by the NHSA

Note: 1. Logic for Administrative Hierarchy-Policy Strength Linkage: Rooted in China’s bureaucratic system – higher hierarchy means stronger legal authority, more resource support, and better implementation effectiveness; multi-agency documents enhance cross-sectoral synergy. 2. Scoring Examples: State Council documents (5 points, e.g. Policy No.10), NHSA-NHC joint documents (4 points, e.g. Policy No.17), NHC independent documents (3 points, e.g. Policy No.9). 3. Multi-Agency Rules: Cross-hierarchy documents score by the highest level; same-hierarchy joint documents retain the corresponding score

Policy intensity is an important indicator measuring the strength and effect of policy implementation, representing its influential force and legal effect. Policy intensity is quantified by administrative hierarchy and document type, with core logic: higher hierarchy endows policies with stronger authority and resource support, while multi-agency collaboration boosts synergy. Based on the administrative level of the issuing body and policy text type, the intensity of HVMC VBP policies was classified into five levels from high to low, enhancing quantification accuracy: Policies/regulations/notices issued by the State Council (5 points); Policy documents jointly issued by multiple departments (4 points); Policy documents issued by the National Health Commission (3 points); Decisions/opinions issued by the NHSA (2 points); Notices issued by the NHSA (1 point).

Policy measures refer to targeted actions taken for main tasks, reflecting specific implementation methods. To address operational ambiguity and ensure scoring consistency, the policy measures scoring standards were optimised to be fully operational (see Supplemental Table S2). Each score (1–5) is anchored to specific, verifiable indicators (e.g. ‘explicit time nodes' for planning measures, ‘unique product codes’ for supervision measures). Intermediate scores (2 and 4) are defined as gradient transitions between adjacent integer scores, ensuring uniform scoring logic across the four measure categories.

To enhance scoring consistency, the 9 cross-disciplinary experts participated in pre-scoring training and group discussions for ambiguous policy clauses, ensuring a shared understanding of the operational criteria. Detailed operational scoring criteria for policy measures (including intermediate score anchors) are provided in Supplemental Table S2.

Policy objectives are the expected purposes, requirements, and results of policy implementation. To address consistency and operability issues, the policy objectives scoring standards were standardised (see Supplemental Table S3). All criteria are strictly based on policy text content (excluding implementation outcomes) and aligned around three uniform dimensions: clarity of core elements, quantifiability of requirements, and completeness of supporting clauses. Intermediate scores (2 and 4) are defined as gradient transitions between adjacent integer scores – score 4 reflects ‘core elements complete but lack quantifiable targets/supporting clauses', while score 2 indicates ‘only vague mention of single core elements without additional details'. Normative assumptions in scoring (e.g. ‘quantifiable requirements reflect higher policy clarity') are consistent with mainstream policy text analysis norms, ensuring objective and reproducible evaluation. The detailed scoring standards are provided in Supplemental Table S3 for reference.

### 2.5. Quantification method

Based on the policy coordination quantification analysis model proposed by Peng Jisheng (Peng et al., 2008), the overall coordination degree of HVMC VBP policies was calculated using the following formulas. The essential influence of policy force can be seen as the product of policy intensity and the score of policy measures or objectives. Therefore, Formula (1) calculates the annual policy force value for China's HVMC VBP policies:

(1) $$\mathrm{TP}G_{i}\;=\;\sum_{\;j=1}^{N}PG_{\mathrm{ij}}\;\times \;P_{\mathrm{ij}}\ldots i\in [2012,\;2024]$$

In Formula (1):

- i represents the year, specifically the years from 2012 to 2024 in this study;
- N represents the total number of centralised procurement policies for high-value medical consumables issued in the year i;
- j represents the j-th policy related to centralised procurement of high-value medical consumables issued in year i, ranging from 1 to N;
- PGij represents the score of each policy goal and policy measure of the j-th policy in year i. When calculating the annual policy intensity, PGij = 1;
- Pij represents the policy intensity score of the j-th policy in year i;
- TPGi represents the overall status of policy goals and policy measures of centralised procurement policies for high-value medical consumables in year i, i.e. the annual policy effectiveness value.

Second, this study draws on the mathematical formulas in Peng Jisheng’s model and modifies them (Formulas 2 and 3) to conduct a comprehensive quantitative analysis of the goal coordination degree and measure coordination degree of centralised procurement policies for high-value medical consumables each year from a macro perspective. This allows for a more accurate measurement and analysis of the coordination effect of centralised procurement policies for high-value medical consumables, and a macro-level study of the overall coordination effect of policies, thereby providing solid data support and theoretical basis for the continuous optimisation of policies.

(2) k≠l

(3) s≠t

In the formulas:

- PMC_i_ represents the policy measure coordination degree of centralised procurement policies for high-value medical consumables in year i;
- PGC_i_ represents the policy goal coordination degree of centralised procurement policies for high-value medical consumables in year i;
- N represents the total number of centralised procurement policies for high-value medical consumables issued in year i;
- pe_j_ represents the policy intensity score of the j-th policy;
- pg_jk_ and pg_jl_ represent the scores of the k-th and l-th policy measures in the j-th policy, where k and l (k≠l) represent two measures selected from the four policy measures (planning and guiding measures, administrative management measures, regulatory safeguard measures, optimising and improving measures) to consider policy measure coordination;
- pm_js_ and pm_jt_ represent the scores of the s-th and t-th policy goals in the j-th policy, where s and t (s≠t) represent two goals selected from the four policy goals (reducing procurement costs and patient burden, improving quality and ensuring supply, optimising price formation and medical insurance payment mechanisms, promoting industrial innovation and industry development) to consider policy goal coordination.

## 3. Results

### 3.1. Descriptive analysis and word frequency

Based on the number of policies issued annually from 2004 to 2024, the development of HVMCs VBP policies was categorised into three distinct periods. The criteria for demarcation were quantitative trends in issuance, key policy events, and stated shifts in reform emphasis within the policy texts.

**Figure 1.**
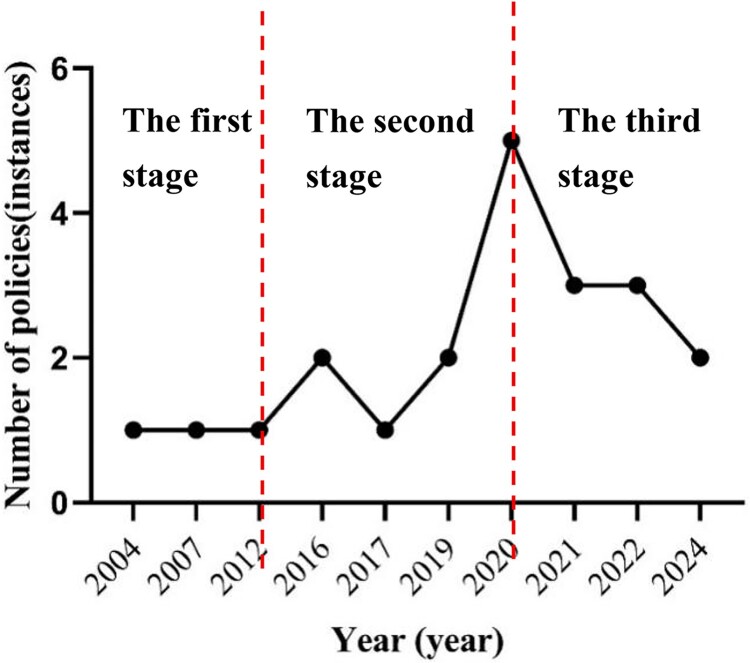
Number of HVMC VBP policies issued annually from 2004 to 2024.

Figure 1 illustrates the annual policy count. The first period (2004–2012) was marked by relatively few policy releases, focused primarily on establishing foundational procurement frameworks. The second period (2012–2020) showed a noticeable increase in policy volume, with an observed shift in terminology towards management and coordination terms. The third period (2020–2024) exhibited fluctuations in annual counts, accompanied by a change in thematic focus from expansion to quality-related aspects.

Word frequency analysis was conducted to identify prominent terms across the three periods (Supplemental Table S4). The terms ‘medical' and ‘institution' were consistently among the most frequent across all periods. The term ‘management' appeared with greater relative prominence in the second period, while ‘winning bid' was observed as a more frequent term in the third period. References to ‘enterprise' and ‘medical insurance' increased notably in frequency during the second and third periods.

### 3.2. Policy intensity analysis

Among the 21 policies, the NHSA and multi-ministry joint issuances accounted for the largest share (24% each) of issuing bodies (Supplemental Figure S1). The State Council issued 3 documents, and the State Council General Office issued 4.

**Figure 2.**
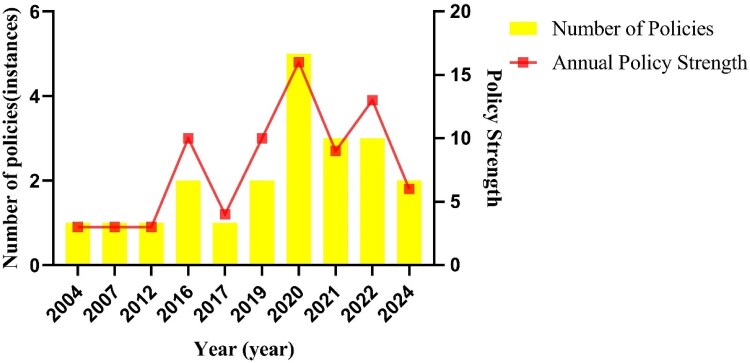
Trends in policy quantity and total policy intensity. Note: Policy total intensity is an objectively aggregated indicator (sum of policy intensity scores), while policy quantity is raw count data; both are non-subjective metrics to avoid misleading correlation interpretations.

Based on the scoring scale described in Section 2.4, policy intensity was scored by the expert panel. The aggregated annual total policy intensity was calculated and is presented alongside the annual policy count in Figure 2.

Figure 2 shows that total policy intensity exhibited an upward trend with fluctuations, reaching its highest aggregate value in 2020. While the number of policies increased in 2020, the average intensity score per policy for that year was lower relative to adjacent years. Average intensity scores showed a recovery in the 2021–2024 period.

### 3.3. Policy measures coordination analysis

**Figure 3.**
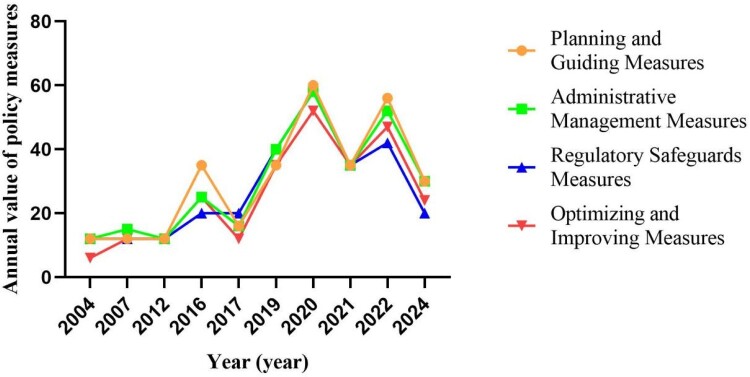
Annual values of policy measures for HVMC VBP policies (line chart). Note: Synergy degrees between pairwise measures (derived from calculated Delphi scores) are provided in Supplemental Figures S2–S5.

Based on the policy measures scoring scale, the expert panel proceeded to score the policy measures. The results are shown in Table S5. The annual values for the four types of policy measures were then calculated using Formula (1), as shown in Figure 3.

Regarding the coordination between measure pairs (calculated via Formula 2 and depicted in Supplemental Figures S2–S5), distinct patterns were observed. The coordination values are constructed indices intended to reflect relative trends rather than absolute magnitudes.

- Coordination between Planning and Guidance Measures and Administrative Management Measures was comparatively higher and more stable than other pairings across the time series. The calculated index for this pairing showed a general increase from the initial period to a peak around 2019, followed by a moderation.
- Coordination values involving Optimisation and Improvement Measures and Regulatory Safeguard Measures exhibited a wider range of fluctuation over the study period, with a notable peak observed in 2019.

### 3.4. Policy objectives scoring results

**Figure 4.**
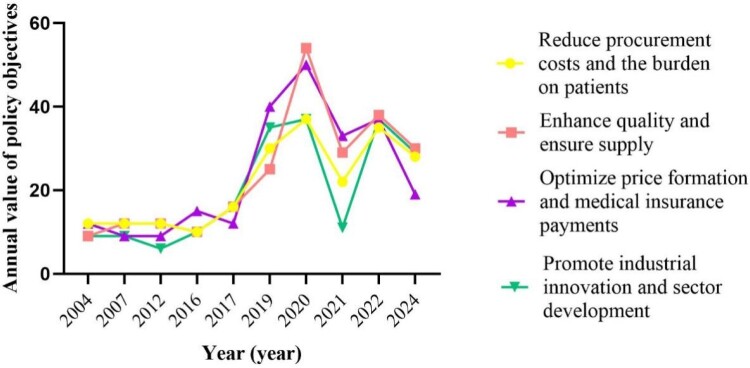
Line chart of annual values of policy goals for centralised procurement of high-value medical consumables. Note: Synergy degrees between pairwise objectives (derived from calculated Delphi scores) are provided in Supplemental Figures S6–S9.

Based on the policy objectives scoring scale, the expert panel proceeded to score the policy objectives. The results are shown in Table S6. The annual values for the four types of policy objectives were then calculated using Formula (1), as shown in Figure 4.

The coordination indices for pairs of objectives (calculated via Formula 3; Supplemental Figures S6–S9) showed distinct temporal patterns. As with the measure indices, these are constructed values intended to indicate relative changes in policy alignment.

- In the first period (2004–2016), calculated coordination values were generally lower across most objective pairs.
- A substantial increase in calculated coordination was observed across multiple objective pairs during the middle period (2017–2020).
- The final period (2021–2024) was characterised by diverging trends among objective pairs. For instance, the calculated index for the pairing of ‘Reducing Costs' and ‘Promoting Innovation' increased to a peak in 2020 and subsequently declined to a lower level by 2024.

### 3.5. Cross-Sectoral implications of policy synergy

Potential implications for medical industry innovation, health insurance financing, and system-wide efficiency were inferred from the coordination patterns of policy measures and objectives. A detailed analysis of these observed patterns is presented in the Discussion section.

## 4. Discussion

### 4.1. Evolution mechanism of policy synergy guided by policy coordination theories

China's HVMC VBP policy has gone through three stages: initial exploration (2004–2012), gradual development (2012–2020), and stable optimisation (2020–2024). The synergy degree exhibits an evolutionary pattern characterised by early dispersion, a mid-term leap, and later differentiation. These patterns are driven by three core policy coordination theories: path dependency, policy layering, and adaptive governance. The reported coordination values are constructed indices and should be interpreted as reflecting relative trends rather than exact measurements.

In the early stage (2004–2012), path dependency contributed to low synergy. Policies focused on building basic procurement frameworks (e.g. pilot centralised procurement), but administrative inertia and unclear cross-departmental responsibilities limited collaboration. This aligns with the research of Wang and Zhang (2017) on new energy vehicle policy synergy, which notes that ‘initial reform policies often face coordination barriers due to solidified institutional habits and departmental boundaries’. The mid-stage (2012–2020) saw a synergy leap driven by policy layering: more joint policies by multiple departments (e.g. NHSA and Ministry of Industry and Information Technology) filled gaps left by single-department policies, pushing total policy intensity to rise sharply. This is consistent with the finding of Zhang et al. (2025) in Beijing-Tianjin-Hebei technology policy research that ‘multi-agency collaboration significantly improves policy synergy efficiency’. In the late-stage (2020–2024), adaptive governance became prominent: reforms shifted from ‘expanding procurement scope' to ‘improving quality', but uneven responses to innovation incentives and supply stability led to differentiated synergy. It should be noted that some year-to-year fluctuations during this stage may reflect model sensitivity rather than substantive policy changes. The recovery of average policy intensity reflects targeted adjustments, echoing the adaptive governance logic described in (OECD, 2024)’s *Medical Supply Chain Coordination Report*.

### 4.2. Key characteristics of policy measures coordination

Among the four policy measures, Planning and Guidance Measures and Administrative Management Measures maintained high and stable synergy. Their coordination values showed a consistent upward trend, which reflects a closed-loop dynamic of ‘top-level design plus implementation supervision’: Planning Measures clarify procurement rules, while Administrative Measures ensure inter-departmental implementation.

However, this dominance appears to have constrained the synergy space available for other measures. Regulatory Safeguard Measures and Optimisation and Improvement Measures exhibited low and volatile coordination. This pattern is consistent with a longstanding emphasis on ‘cost reduction' over ‘quality and supply optimization', as noted in the research of Xie and Liu (2016) on medical consumables procurement.

### 4.3. Core contradictions in policy objectives coordination

The core contradiction in policy objectives is between ‘cost control' and ‘industrial innovation'. In the early stage, path dependency led to a predominant focus on cost reduction (e.g. 30% price cuts for HVMCs), resulting in low synergy between innovation and other objectives. This is in line with Gao and Liang's (2019) research on local government policies: ‘Single policy objectives often cause fragmented coordination, as departments prioritise their core tasks while ignoring cross-objective collaboration’.

In the mid-stage, policy layering expanded objectives to include medical insurance payment and quality assurance (e.g. linking procurement prices to insurance payment standards), thereby enhancing synergy between cost reduction and price optimisation.

In the late-stage, synergy between quality improvement and innovation strengthened, but gaps remained: issues such as delayed medical insurance fund allocation and mismatched payment standards persisted. Unlike Western countries (e.g. the UK) that balance innovation and cost through health technology assessment (HTA), China’s VBP framework currently lacks a mature innovation incentive mechanism.

### 4.4. Cross-sectoral implications of policy synergy

#### 4.4.1. Medical industry innovation

Low early synergy and a narrow focus on cost-control objectives likely reduced enterprise innovation incentives, as the ‘volume-for-price' mechanism prioritised market share over high-risk R&D. Although late-stage synergy between quality and innovation objectives improved, the persistently low coordination of Optimisation and Improvement Measures limited the effectiveness of incentives. For instance, a lack of preferences for innovative products and insufficient R&D subsidies hindered sustainable innovation.

#### 4.4.2. Health insurance financing

The relatively high-synergy between cost reduction and price-payment optimisation effectively contributed to reducing medical insurance expenditure via bulk procurement. Early misalignment between medical insurance and procurement systems caused fund allocation delays, but the subsequent increase in multi-department high-intensity policies and the rising frequency of the keyword ‘medical insurance' indicated improved financing coordination, thereby enhancing fund stability.

#### 4.4.3. System-wide efficiency

High coordination between Planning and Guidance and Administrative Management Measures facilitated inter-departmental collaboration, while weak synergy between Optimisation and Improvement and Regulatory Safeguard Measures was associated with inefficiencies (e.g. delayed product adoption, procurement volume mismatches). Post-2021 quality-oriented policies and recovered average intensity contributed to improved resource allocation and reduced inefficiencies, thereby boosting overall system efficiency.

### 4.5. Linkage with policy recommendations and study limitations

Key problems identified via the framework’s diagnostic function – unbalanced measure coordination, cost-innovation contradictions, insufficient cross-sectoral linkage – directly inform the policy recommendations. Strengthening top-level coordination addresses inter-departmental inertia, enhancing implementation flexibility resolves supply-quality mismatches, and innovation incentives mitigate cost-innovation tensions. These recommendations align with the framework’s priority-setting guidance, promoting transformation to a ‘multi-dimensional coordinated' VBP system.

This study has limitations: no robustness or sensitivity tests were conducted, so adjustments to scoring ranges, indicator weights, or policy samples may affect synergy degree stability. Future research should address this by refining quantitative methods and expanding policy samples. Additionally, the policy synergy matrix (Table 2) visualises interactions among the three-dimensional framework, aiding interpretation of how policy intensity, measures, and objectives jointly shape synergy outcomes.

**Table 2.** Policy synergy matrix of three-dimensional framework.

Interaction dimension	Description of interaction mechanism	Outcome of synergy
PI × PM	Higher policy intensity strengthens measure implementation; multi-department policies promote cross-measure coordination	High-intensity policies drive strong measure synergy (e.g. Planning and Administrative); low-intensity policies limit effectiveness
PI × PO	High-intensity policies prioritise core objectives; State Council documents promote balanced objective synergy in later stages	Early high-intensity policies exacerbated imbalance; later ones improved coordination (e.g. quality + innovation)
PM × PO	Planning measures support cost reduction/price optimisation; administrative measures enhance quality/supply; optimisation measures promote innovation	High-synergy measures drive core objectives; weak synergy limits comprehensive realisation

### 4.6. Research assumptions and limitations

#### 4.6.1. Research assumptions

This study is based on three verifiable assumptions:

1. The three-dimensional framework (intensity-measures-objectives) comprehensively captures HVMCs policy synergy, verified by expert consensus;
2. National-level policy texts accurately reflect policy intentions (formal documents from central agencies have clear regulatory functions);
3. Expert scoring (1–5 scale) objectively reflects policy attributes (cross-disciplinary experts avoid single-perspective bias).

#### 4.6.2. Limitations

This study has four limitations:

1. No robustness or sensitivity tests were conducted. The calculated synergy degree values may therefore vary with changes in scoring ranges, indicator weights, or policy sample sizes. Readers and policymakers should interpret the numerical results as relative trends rather than absolute measurements;
2. The sample only includes 21 national-level policies, excluding local policies (may ignore regional coordination differences);
3. Text mining relies on keyword frequency, which may miss implicit policy intentions (e.g. unstated innovation incentives);
4. The study focuses on policy design rather than implementation effects (synergy scores reflect theoretical coordination, not real-world execution).
5. Some scoring decisions remain normative (e.g. higher scores for quantifiable targets). This may affect interpretation – for instance, a policy with strong quantitative targets may score high despite weak implementation. Thus, findings should be used as diagnostic indicators, not definitive rankings.

Future research will expand local policy samples and conduct robustness tests to address these limitations.

### 4.7. Synthesis of results, model boundaries, and policy context

#### 4.7.1. Synthesis of core findings

This study’s results collectively reveal the evolutionary logic of HVMC VBP policy synergy in China: from early dispersion driven by path dependency, to mid-term leap enabled by multi-department collaboration, to late differentiation due to adaptive governance focusing on quality improvement. Key patterns include high stable synergy between Planning and Administrative Measures, low volatile synergy between Optimisation and Regulatory Measures, and persistent contradictions between cost-control and industrial innovation objectives. These findings collectively indicate that China’s VBP reform has achieved progress in cost reduction but needs systemic optimisation in multi-dimensional coordination.

#### 4.7.2. What the model can and cannot measure

- **What it can measure**: The three-dimensional framework and synergy model effectively capture relative trends of policy synergy across intensity, measures, and objectives. It quantifies the alignment between policy design elements (e.g. whether cost-control objectives are coordinated with medical insurance payment measures) and reflects cross-stage evolutionary characteristics.
- **What it cannot measure**: First, modelled outputs (quantified coordination degrees) differ from real-world administrative coordination: The former is derived from policy text analysis and expert scoring, focusing on ‘design-level alignment'; the latter involves practical factors such as inter-departmental communication efficiency, local implementation capacity, and stakeholder collaboration – factors not captured by text-based modelling. Second, the model does not capture implicit policy intentions or regional differences, as local policies are excluded from the sample. Third, year-wise fluctuations in coordination values may reflect model sensitivity.

#### 4.7.3. Contextualisation in policy practice

The findings are closely aligned with China’s healthcare reform practice. The mid-term synergy leap corresponds to the promotion of ‘Healthy China 2030' and multi-department joint governance. The late focus on quality improvement echoes the deepening of Sanming Healthcare Reform, which emphasises balancing cost control and industrial development. The cost-innovation contradiction reflects the practical challenge of reconciling short-term people’s livelihood needs (reducing patient burden) and long-term industrial competitiveness (promoting domestic HVMC innovation). These contextual links highlight the study’s practical relevance for policy optimisation.

#### 4.7.4. Practical implications for policy makers

This study’s three-dimensional framework and findings serve policy makers as a diagnostic, monitoring, and priority-setting tool.

Diagnostic tool: Identifies specific coordination gaps (e.g. weak optimisation-supervision synergy, cost-innovation contradictions) via quantifiable indicators.

Monitoring tool: Tracks dynamic synergy changes through PMC_i_/PGC_i_ indicators to assess reform effectiveness timely.

Priority-setting tool: Guides prioritising key tasks (e.g. strengthening insurance-policy linkage, balancing cost-control and innovation).

These applications translate theoretical findings into actionable support for evidence-based policy making.

## 5. Conclusion

This study demonstrates that the evolution of China's HVMC VBP policy documents reflects a process of increasing complexity but not necessarily uniformly increasing coordination. Importantly, this study assesses policy text coherence – it measures expert-weighted indicator consistency and models policy coordination based on policy texts, expert scoring, and quantitative analysis. Notably, there is no evidence related to the measurement of interagency cooperation, implementation quality, or institutional capacity in this research. The observed lack of synergy, particularly between cost-control measures and innovation-promotion objectives, represents a design-level consideration for the long-term sustainability of the reform.

A limitation of this study is the absence of robustness or sensitivity tests, which means adjustments to scoring ranges, indicator weights, or policy sample sizes may affect the stability of the calculated synergy degree. This limitation is disclosed to ensure research transparency.

Future policy optimisation could benefit from moving beyond a single-dimensional focus on price reduction towards a multi-dimensional approach that dynamically balances cost, quality, access, and innovation. Future research may address this limitation by conducting robustness and sensitivity tests (e.g. adjusting scoring scales or expanding the policy sample) to enhance result stability. The methodologies and frameworks developed herein may also be applied to evaluate policy coordination in other healthcare domains and beyond, subject to appropriate contextual adaptation.

## 6. Policy recommendations

Based on the analysis, the following integrated policy recommendations are proposed to enhance the coordination and effectiveness of China’s volume-based procurement system for high-value medical consumables:

### Strengthen top-level policy coordination

A multi-tiered, cross-departmental coordination mechanism should be established under the leadership of the State Council. This includes formulating a ‘1 + N' policy framework to enhance inter-ministerial collaboration and systemic governance, addressing the low and highly volatile synergy between Optimisation and Improvement Measures and Regulatory Safeguard Measures observed in the results (characterised by a low initial value, a sharp peak around 2019, and a subsequent decline). A pre-policy assessment mechanism involving experts from healthcare, insurance, industry, and regulatory agencies should be introduced to evaluate potential coordination challenges and simulate policy effects prior to implementation. Furthermore, clarifying functional responsibilities across agencies – such as the National Healthcare Security Administration’s pricing authority and the State Administration for Market Regulation’s regulatory mandates – is critical to reducing ambiguities and improving accountability, which responds to the cross-departmental coordination gaps reflected in the ‘late-stage differentiation' trend of policy synergy in the discussion.

### Enhance implementation flexibility and feedback integration

To mitigate the slow adoption of centralised procurement results and mismatch between volume commitments and actual use, a more flexible execution system is recommended. This includes implementing trial periods for new products entering the procurement catalog, improving classification-based guidance for patients and training programs for medical personnel, and establishing dual-channel feedback from both physicians and patients to enable rapid policy refinement, targeting the insufficient adaptation between medical insurance payment and industry development, as well as the sharp fluctuations in partial policy measures noted in the results (for instance, the coordination between Optimisation and Improvement Measures and Regulatory Safeguard Measures exhibited a marked decrease from 2020 to 2021). Additionally, a dynamic assessment system that incorporates real-world safety data and adverse event monitoring should be developed. Hospitals could be allowed limited autonomy to procure non-winning products after meeting primary procurement targets, thus increasing clinical adaptability without compromising bulk procurement objectives, responding to the gap between policy design and practical implementation needs reflected in the results.

### Foster innovation through incentives and insurance linkages

To alleviate the tension between cost reduction and innovation sustainability, targeted measures should be introduced. These include establishing special innovation funds and R&D subsidies to support enterprises in high-end consumable development, along with protection periods that temporarily exempt innovative domestic products from VBP inclusion to ensure market returns and encourage investment, directly addressing the persistent contradiction between cost-control and industrial innovation indicated by the coordination trends (values increased from a low level to a peak around 2020 before moderating by 2024). Medical insurance policies must be synergistically aligned, with dedicated payment channels for innovative products, value-based tiered reimbursement, and increased reimbursement ratios to encourage uptake, responding to the insufficient synergy between medical insurance payment and industrial development highlighted in the discussion. An annual evaluation mechanism should be instituted to dynamically optimise payment standards and foster long-term industry development.

**Figure 5.**
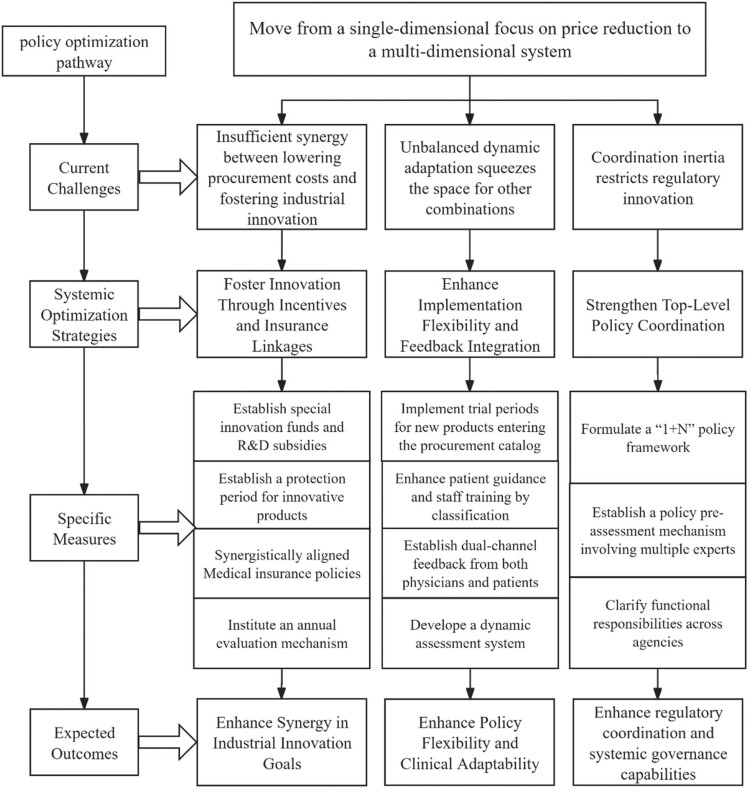
Policy optimisation roadmap.

These recommendations, summarised in Figure 5, aim to transform China’s VBP system from a volume-price-driven model into a coordinated policy regime that balances cost efficiency, quality assurance, supply stability, and sustainable innovation.

## Supplementary Material

Supplemental Material
