# Supplementary material for: Systemic evaluation and optimisation strategies for the synergy of high-value medical consumables policies in China
Source: J Pharm Policy Pract. 2026 Jul 31;19(1):2673696. doi: 10.1080/20523211.2026.2673696 (PMC13431012; doi:10.1080/20523211.2026.2673696)
Supplement: Supplemental Material [file JPPP_A_2673696_SM3310.docx]

**Supplementary Material**

**Table S1 Summary of National-Level Policies Related to HVMC VBP**

*Selection Logic: This table includes 21 national-level policies issued during 2004–2024. Inclusion criteria: ① Issued by central authorities (Party Central Committee, State Council, national ministries/commissions); ② Directly related to HVMC volume-based procurement (VBP) with explicit objectives/measures; ③ Formal documents (plans, opinions, notices, standards) with complete content. Exclusion criteria: ① Local implementation rules, reply letters, or drafts; ② Policies unrelated to HVMC VBP or lacking specific operational requirements. 2. Document Type Definition: ① Strategic Plan: Medium-to-long-term policy frameworks guiding overall reform direction; ② Technical Notice: Specific implementation documents for special tasks, supervision, or coordination; ③ Regulatory Standard: Documents defining operating procedures, evaluation criteria, or management rules for VBP. 3. Terminology Standardization: Government department names adopt official full/standardized abbreviations (e.g., "National Healthcare Security Administration" = NHSA, first full name then abbreviation); avoid ambiguous expressions like "and other departments" by listing all involved authorities.*

| No. | Issue Date | Issuing Department (Standardized) | Document Title | Document Type |
| --- | --- | --- | --- | --- |
| 1 | 2004/8/31 | Ministry of Health (former) | Pilot Work Plan for Centralized Procurement of High-Value Medical Consumables in Medical Institutions of 8 Provinces/Cities | Technical Notice |
| 2 | 2007/6/21 | Ministry of Health (former) | Notice on Further Strengthening the Management of Medical Device Centralized Procurement | Technical Notice |
| 3 | 2012/12/17 | National Health and Family Planning Commission (former) | Work Specifications for Centralized Procurement of High-Value Medical Consumables (Trial) | Regulatory Standard |
| 4 | 2016/10/25 | CPC Central Committee, State Council | "Healthy China 2030" Plan Outline | Strategic Plan |
| 5 | 2016/12/27 | CPC Central Committee, State Council | Notice on Printing and Distributing the "13th Five-Year Plan" for Deepening the Reform of the Medical and Health System | Strategic Plan |
| 6 | 2017/7/11 | General Office of the National Development and Reform Commission, General Office of the Ministry of Industry and Information Technology, General Office of the Ministry of Finance, General Office of the National Health and Family Planning Commission, General Office of the China Food and Drug Administration, General Office of the State Administration of Traditional Chinese Medicine, State Council Office for Correcting Unhealthy Trends in Professions | Notice on Printing and Distributing the Special Rectification Activity Plan for Medical Consumables | Technical Notice |
| 7 | 2019/7/19 | General Office of the State Council | Notice on Printing and Distributing the Reform Plan for Governing High-Value Medical Consumables | Strategic Plan |
| 8 | 2019/11/18 | State Council Leading Group for Deepening the Reform of the Medical and Health System | Notice on Further Promoting the Experience of Deepening the Reform of the Medical and Health System in Fujian Province and Sanming City | Technical Notice |
| 9 | 2020/1/14 | National Health Commission | Notice on Printing and Distributing the First Batch of National Key Governance Lists for High-Value Medical Consumables | Technical Notice |
| 10 | 2020/2/25 | State Council | Opinions on Deepening the Reform of the Medical Security System | Strategic Plan |
| 11 | 2020/7/16 | General Office of the State Council | Notice on Printing and Distributing the Key Work Tasks for Deepening the Reform of the Medical and Health System in the Second Half of 2020 | Technical Notice |
| 12 | 2020/11/20 | Medical Price and Bidding Procurement Guidance Center of the National Healthcare Security Administration | Notice on Printing and Distributing the Discretionary Benchmarks for Medical Price and Bidding Procurement Credit Rating (2020 Edition) | Regulatory Standard |
| 13 | 2020/12/17 | National Healthcare Security Administration (NHSA) | Opinions on Supporting Measures for the Centralized Volume-Based Procurement and Use of Coronary Stents Organized by the State | Technical Notice |
| 14 | 2021/6/4 | National Healthcare Security Administration, National Development and Reform Commission, Ministry of Industry and Information Technology, Ministry of Finance, Ministry of Human Resources and Social Security, National Health Commission, National Medical Products Administration, State Administration of Traditional Chinese Medicine | Opinions on Carrying Out the Centralized Volume-Based Procurement and Use of High-Value Medical Consumables Organized by the State | Strategic Plan |
| 15 | 2021/6/8 | National Healthcare Security Administration (NHSA), Ministry of Finance, State Taxation Administration | Notice on Doing a Good Job in Basic Medical Security for Urban and Rural Residents in 2021 | Technical Notice |
| 16 | 2021/8/30 | Medical Price and Bidding Procurement Guidance Center of the National Healthcare Security Administration | Notice on Printing and Distributing the List of Public Service Items for the Centralized Procurement of Drugs and Medical Consumables | Regulatory Standard |
| 17 | 2022/3/31 | General Office of the National Healthcare Security Administration, General Office of the National Health Commission | Opinions on Supporting Measures for the Centralized Volume-Based Procurement and Use of High-Value Medical Consumables (Artificial Joints) Organized by the State | Technical Notice |
| 18 | 2022/5/4 | General Office of the State Council | Notice on Printing and Distributing the Key Work Tasks for Deepening the Reform of the Medical and Health System in 2022 | Technical Notice |
| 19 | 2022/7/8 | National Healthcare Security Administration (NHSA), Ministry of Finance, State Taxation Administration | Notice on Doing a Good Job in Basic Medical Security for Urban and Rural Residents in 2022 | Technical Notice |
| 20 | 2024/5/14 | National Healthcare Security Administration (NHSA) | Notice on Strengthening Regional Coordination and Doing a Good Job in Improving Quality and Expanding Coverage of Medical and Pharmaceutical Centralized Procurement in 2024 | Technical Notice |
| 21 | 2024/5/20 | National Healthcare Security Administration (NHSA) | Notice on Further Promoting the Sanming Medical Reform Experience and Continuously Promoting the Innovative Development of Medical Security Work | Technical Notice |

**Table S2 Operational Scoring Standards for Policy Measures (Supplementary Material)**

*Note: 1. Operational Criteria Design: Each measure’s scoring is based on 3 core operational indicators (clarity of requirements, completeness of content, feasibility of implementation) to avoid descriptive ambiguity. 2. Intermediate Score Anchors: Scores 2 and 4 are defined as gradient transitions between adjacent integer scores (1→3→5), ensuring consistent scoring logic across categories. 3. Scoring Consistency Assurance: The 9-member cross-disciplinary expert panel conducted pre-scoring training and group discussions for ambiguous clauses, ensuring uniform understanding of scoring standards.*

| Policy Measure | Score | Operational Scoring Criteria |
| --- | --- | --- |
| Planning and Guidance Measures | 5 | Clearly defines procurement scope (e.g., specific HVMC categories), detailed implementation rules (e.g., bidding procedures), and a ≥3-year medium-term plan with explicit time nodes (e.g., "complete regional alliance procurement by 2025"). |
|  | 4 | Clearly defines procurement scope and detailed rules, but lacks medium-term planning or time nodes. |
|  | 3 | Partially defines procurement scope or rules (e.g., only lists categories without bidding rules), with incomplete content. |
|  | 2 | Only vaguely mentions one core element (either scope, rules, or planning), without specific details. |
|  | 1 | No relevant content or extremely vague descriptions (e.g., only "promote centralized procurement" without actionable details). |
| Administrative Management Measures | 5 | Comprehensively specifies organizational structure (e.g., leading group composition), coordination mechanisms (e.g., monthly joint meetings), and promotion responsibilities (e.g., provincial health department as lead), with clear division of labor. |
|  | 4 | Specifies organizational structure and coordination mechanisms, but lacks clear promotion responsibilities. |
|  | 3 | Partially describes organizational structure or coordination mechanisms (e.g., only mentions "establish a leading group" without composition), with incomplete details. |
|  | 2 | Vaguely mentions organizational or coordination elements (e.g., "strengthen inter-departmental coordination" without specific methods), with no actionable content. |
|  | 1 | No relevant content or extremely vague descriptions (e.g., only "strengthen management" without specific measures). |
| Supervision and Safeguard Measures | 5 | Details full-process supervision (e.g., procurement process audit + post-purchase quality sampling), clear penalty clauses (e.g., 2-year ban for supply disruptions), and quality traceability requirements (e.g., unique product codes). |
|  | 4 | Details full-process supervision and penalty clauses, but lacks quality traceability requirements. |
|  | 3 | Partially describes supervision or penalty measures (e.g., only mentions "strengthen quality supervision" without sampling rules), with incomplete content. |
|  | 2 | Vaguely mentions supervision or penalties (e.g., "impose penalties for violations" without specific standards), with no actionable details. |
|  | 1 | No relevant content or extremely vague descriptions (e.g., only "strengthen supervision" without specific measures). |

**Table S3 Operational Scoring Standards for Policy Objectives (Supplementary Material)**

*Note: 1. Standardized Scoring Logic: All criteria are based on policy text content (excluding implementation outcomes) and focus on three core operational dimensions: clarity of core elements, quantifiability of requirements, and completeness of supporting clauses. 2. Intermediate Score Anchors: Scores 2 and 4 are defined as gradient transitions between adjacent integer scores (1→3→5), ensuring consistent application across objectives. 3. Normative Assumption Justification: Scoring logic aligns with policy text analysis norms, prioritizing explicit, actionable content to avoid subjective judgment—core assumptions include "quantifiable requirements reflect higher clarity" and "supporting clauses enhance implementation feasibility."*

| Policy Objective | Score | Operational Scoring Criteria |
| --- | --- | --- |
| Reducing Procurement Costs and Patient Burden | 5 | Explicitly mentions 2+ core elements (circulation streamlining, patient cost reduction, medical insurance surplus retention) + quantifiable targets (e.g., ≥30% price reduction, 30% advance payment) + clear implementation clauses. |
|  | 4 | Explicitly mentions 2 core elements + semi-quantifiable targets (e.g., "significant price reduction" without specific ratio) + basic implementation clauses. |
|  | 3 | Explicitly mentions 1-2 core elements + directional requirements (e.g., "reduce patient burden") + incomplete implementation clauses. |
|  | 2 | Vaguely mentions 1 core element (e.g., only "cost control") + no targets + no implementation clauses. |
|  | 1 | No relevant content or extremely vague descriptions (e.g., only "improve medical accessibility" without cost-related clauses). |
| Improving Quality and Ensuring Supply | 5 | Explicitly mentions 2+ core elements (quality traceability, full-chain supervision, supply stability) + dual-index assessment (quality + supply) + mandatory clauses (e.g., quality insurance, breach accountability). |
|  | 4 | Explicitly mentions 2 core elements + single-index assessment (quality or supply) + non-mandatory clauses (e.g., "encourage quality supervision"). |
|  | 3 | Explicitly mentions 1 core element + directional requirements (e.g., "quality first") + no specific supervision/supply clauses. |
|  | 2 | Vaguely mentions 1 core element (e.g., only "ensure product safety") + no assessment criteria + no clauses. |
|  | 1 | No relevant content or extremely vague descriptions (e.g., only "meet clinical needs" without quality/supply clauses). |
| Optimizing Price Formation and Medical Insurance Payment | 5 | Explicitly mentions 2+ core elements (unified price reference, dynamic adjustment, insurance-procurement linkage) + national-level scope + clear payment standard clauses. |
|  | 4 | Explicitly mentions 2 core elements + regional pilot scope + basic payment linkage clauses. |
|  | 3 | Explicitly mentions 1 core element + directional requirements (e.g., "improve price mechanism") + incomplete linkage clauses. |
|  | 2 | Vaguely mentions 1 core element (e.g., only "rationalize prices") + no scope definition + no linkage clauses. |
|  | 1 | No relevant content or extremely vague descriptions (e.g., only "study payment mechanisms" without specific clauses). |

**Table S4 Word Frequency Statistics for the Three Stages** 1. Weighting Method: Adopts TF-IDF (Term Frequency-Inverse Document Frequency) weighting. Weighted frequency = raw frequency × normalized IDF weight (range: 0.1–1.0), with higher values indicating stronger policy relevance.

2. Stage Division Basis: Determined by policy core functions, reform nodes, and time sequence (Policy 1~21):
Stage 1 (2004–2012, Oldest): "Initial Exploration Stage" – Focus on establishing centralized procurement frameworks and pilot promotion (core tasks: pilot implementation, basic system construction), corresponding to the earliest policies with preliminary reform orientation.

Stage 2 (2013–2020, Middle): "Gradual Development Stage" – Expand procurement scope, strengthen inter-departmental coordination (core tasks: scale expansion, management system improvement), corresponding to policies with improved institutional design.

Stage 3 (2021–2024, Latest): "Stable Optimization Stage" – Focus on quality improvement, medical insurance linkage, and industrial innovation (core tasks: refined management, multi-objective synergy), corresponding to the latest policies with in-depth reform orientation.

3.Document Assignment by Stage (Policy 1~21, 1=latest, 21=oldest):
Stage 1 (2004–2012): Policy 1 (2004), 2 (2007), 3 (2012) [3 documents, oldest 3].

Stage 2 (2013–2020): Policy 4 (2016), 5 (2016), 6 (2017), 7 (2019), 8 (2019), 9 (2020), 10 (2020), 11 (2020), 12 (2020), 13 (2020) [10 documents, middle period].

Stage 3 (2021–2024): Policy 14 (2021), 15 (2021), 16 (2021), 17 (2022), 18 (2022), 19 (2022), 20 (2024), 21 (2024) [8 documents, latest 8].

**Table S2 Word Frequency Statistics for the Three Stages**

| **Stage 1** | | | **Stage 2** | | | **Stage 3** | | | |
| --- | --- | --- | --- | --- | --- | --- | --- | --- | --- |
| No. | Keyword | Freq. | No. | Keyword | Freq. | | No. | Keyword | Freq. |
| 1 | Procurement | 111 | 1 | Procurement | 277 | | 1 | Procurement | 158 |
| 2 | Centralized | 58 | 2 | Medical Consumables | 236 | | 2 | Winning Bid | 90 |
| 3 | Institution | 48 | 3 | Centralized | 187 | | 3 | Medical | 84 |
| 4 | Medical | 38 | 4 | Institution | 162 | | 4 | Centralized | 68 |
| 5 | Pilot | 24 | 5 | Medical | 153 | | 5 | Institution | 64 |
| 6 | Medical Devices | 24 | 6 | High-value | 119 | | 6 | Medical Insurance | 62 |
| 7 | Medical Consumables | 22 | 7 | Management | 104 | | 7 | Centralized Procurement | 61 |
| 8 | High-value | 21 | 8 | Enterprise | 92 | | 8 | Enterprise | 52 |
| 9 | Expert | 19 | 9 | Medical Insurance | 78 | | 9 | National | 51 |
| 10 | Health | 19 | 10 | Department | 50 | | 10 | Medical Consumables | 49 |
| 11 | Committee | 17 | 11 | Service | 47 | | 11 | Implement | 45 |
| 12 | Bidding | 17 | 12 | Pharmaceutica | 45 | | 12 | Service | 41 |

**Table S5 Policy Measures Scores for HVMC VBP Policies**

| **Policy No.** | 1 | 2 | 3 | 4 | 5 | 6 | 7 |
| --- | --- | --- | --- | --- | --- | --- | --- |
| Measure Score | 14 | 17 | 16 | 9 | 12 | 16 | 15 |
| **Policy No.** | 8 | 9 | 10 | 11 | 12 | 13 | 14 |
| Measure Score | 15 | 9 | 17 | 15 | 7 | 17 | 16 |
| **Policy No.** | 15 | 16 | 17 | 18 | 19 | 20 | 21 |
| Measure Score | 16 | 12 | 16 | 13 | 17 | 19 | 17 |

**Table S6 Policy Objectives Scores for HVMC VBP Policies**

| **Policy No.** | 1 | 2 | 3 | 4 | 5 | 6 | 7 |
| --- | --- | --- | --- | --- | --- | --- | --- |
| Goal Score | 14 | 14 | 13 | 4 | 5 | 15 | 15 |
| **Policy No.** | 8 | 9 | 10 | 11 | 12 | 13 | 14 |
| Goal Score | 11 | 6 | 16 | 8 | 12 | 14 | 13 |
| **Policy No.** | 15 | 16 | 17 | 18 | 19 | 20 | 21 |
| Goal Score | 9 | 7 | 16 | 7 | 12 | 16 | 18 |

**Figure S1**


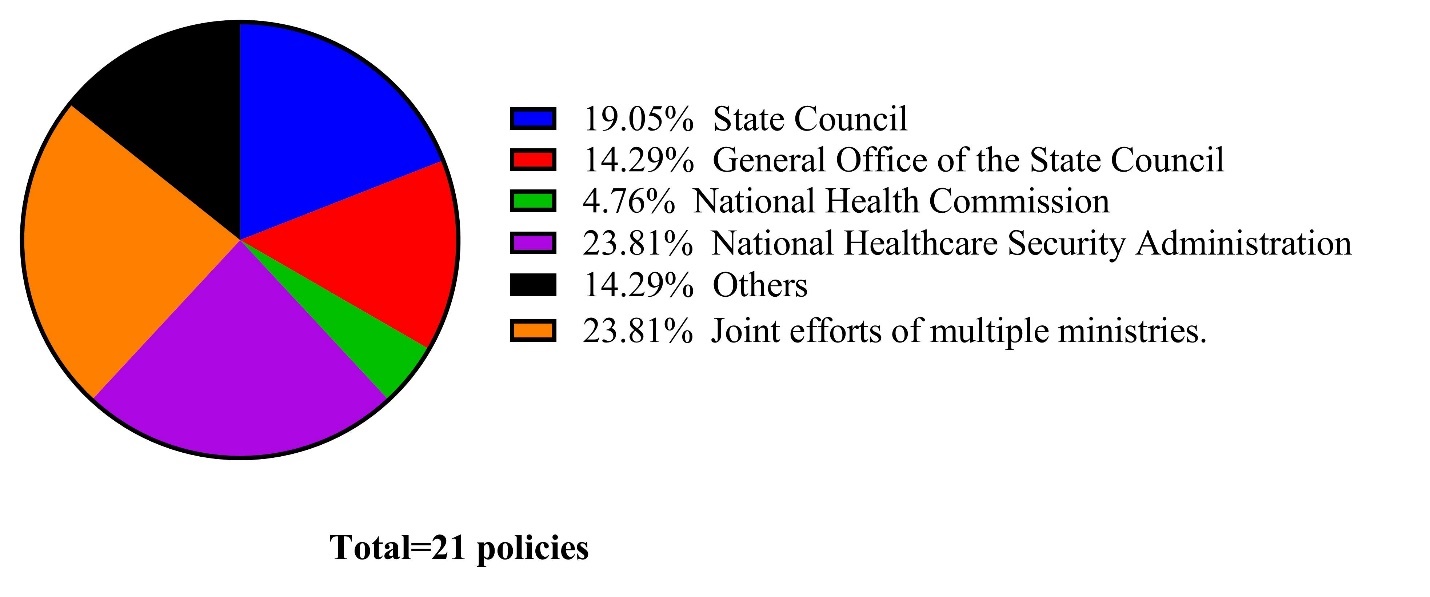
**Figure S1 Issuing Agencies of HVMC VBP Policies**

**Figure S2-S5**

**
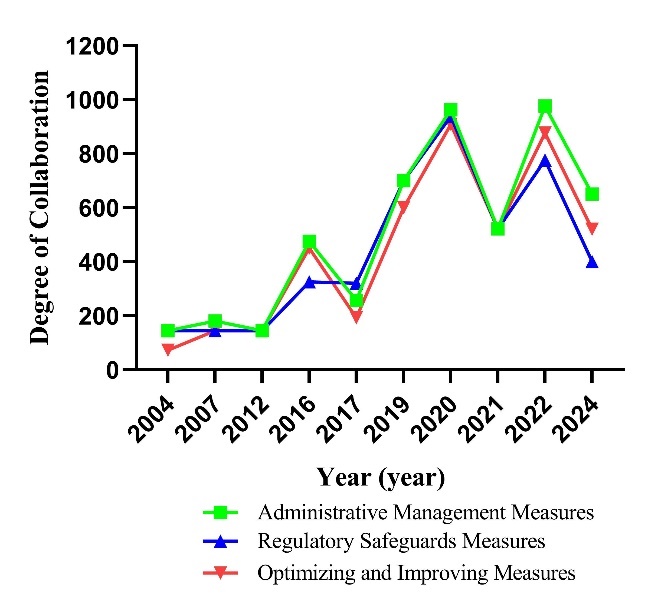
**

**
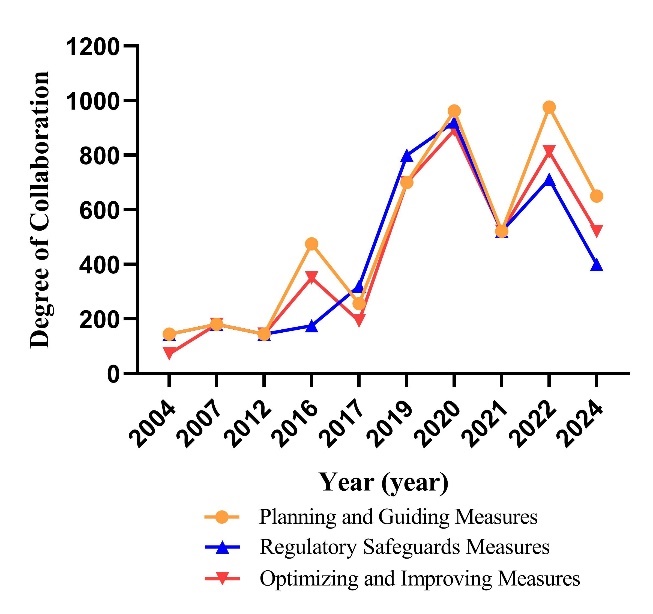
Figure S2 Coordination Degree: Planning & Guidance with Other Measures**

**
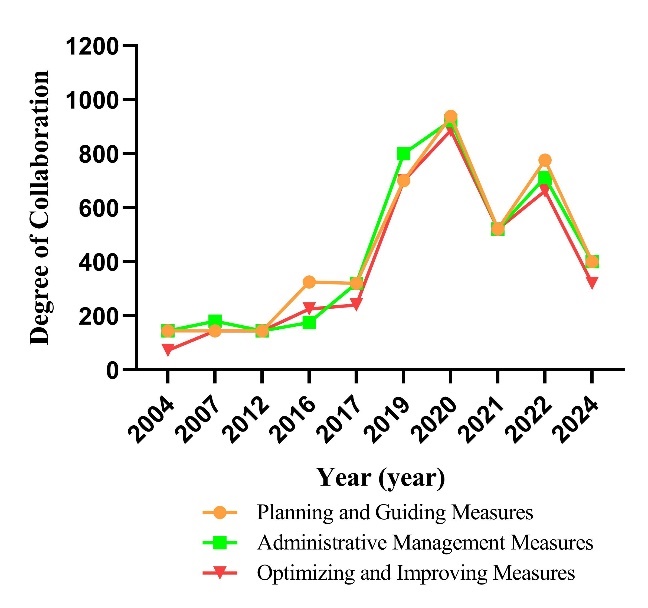
Figure S3 Coordination Degree: Administrative Management with Other Measures**

**Figure S4 Coordination Degree: Supervision & Safeguard with Other Measures**

**
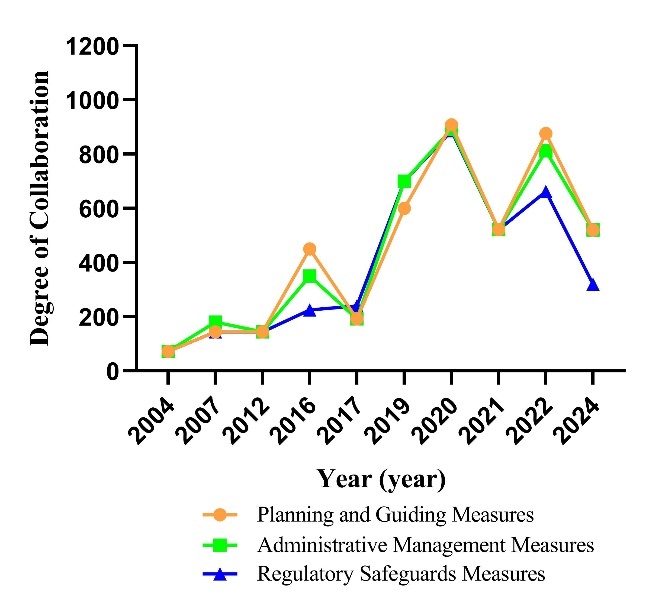
Figure S5 Coordination Degree: Optimization & Improvement with Other Measures**

**Figure S6-S9**


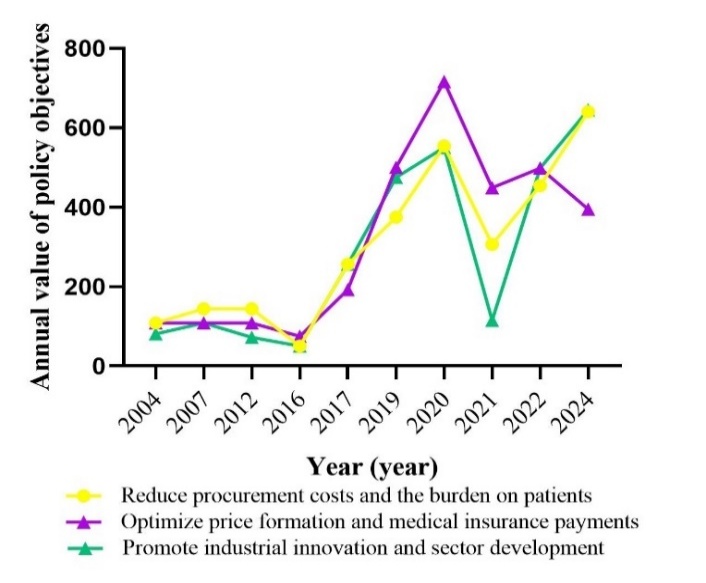


**Figure S6 Coordination Degree: Reduce Cost/Burden with Other Objectives**


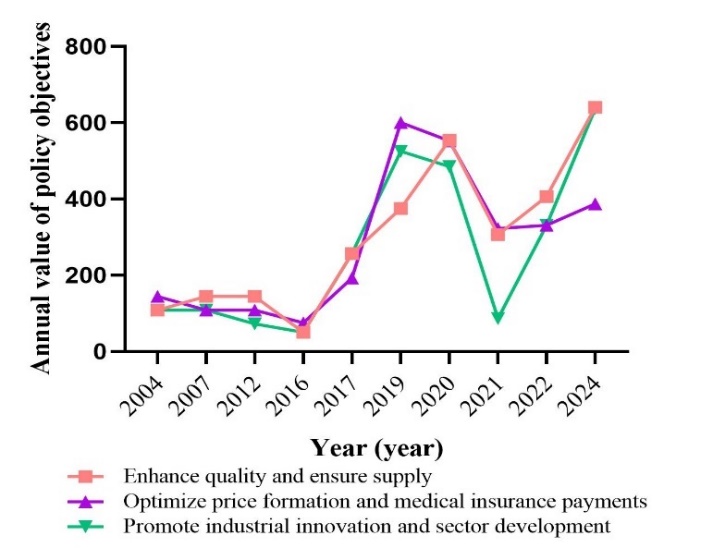


**Figure S7 Coordination Degree: Improve Quality/Supply with Other Objectives**


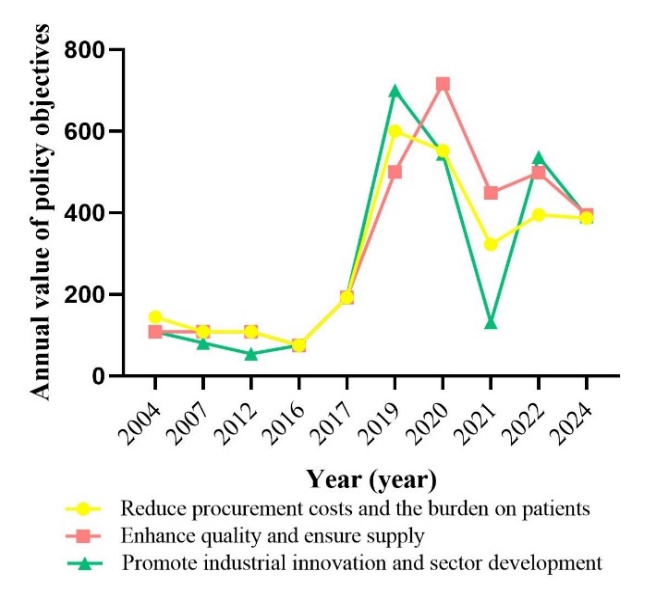


**Figure S8 Coordination Degree: Optimize Price/Payment with Other Objectives**


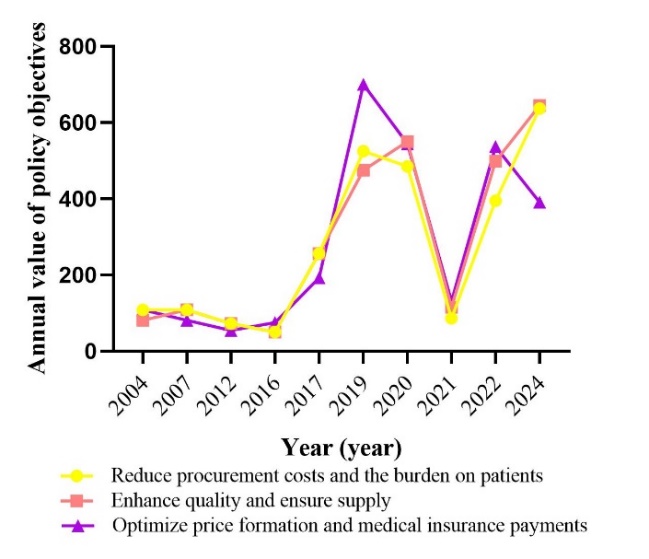


**Figure S9 Coordination Degree: Promote Innovation/Development with Other Objectives**
